# Supplementary material for: Borax induces osteogenesis by stimulating NaBC1 transporter via activation of BMP pathway
Source: Commun Biol. 2020 Nov 27;3:717. doi: 10.1038/s42003-020-01449-4 (PMC7695834; doi:10.1038/s42003-020-01449-4)
Supplement: Supplementary file 1 — Supplementary Information [file 42003_2020_1449_MOESM1_ESM.pdf]

Borax induces osteogenesis by stimulating NaBC1 transporter via activation of BMP pathway

Patricia Rico[1,2*], *Aleixandre Rodrigo-Navarro*[3], *Laura Sánchez Pérez*[2], *Manuel Salmeron-Sanchez*[1,2,3*]

[1] Biomedical Research Networking Center in Bioengineering, Biomaterials and Nanomedicine (CIBER-BBN). 28029 Madrid. Spain.

[2] Center for Biomaterials and Tissue Engineering (CBIT).
Universitat Politècnica de València
Camino de Vera s/n
46022 Valencia, Spain.

[3] Centre for the Cellular Microenvironment
University of Glasgow
Glasgow G12 8LT, United Kingdom.

Supplementary information

Supplementary Figure S1. Cytotoxicity of borax.

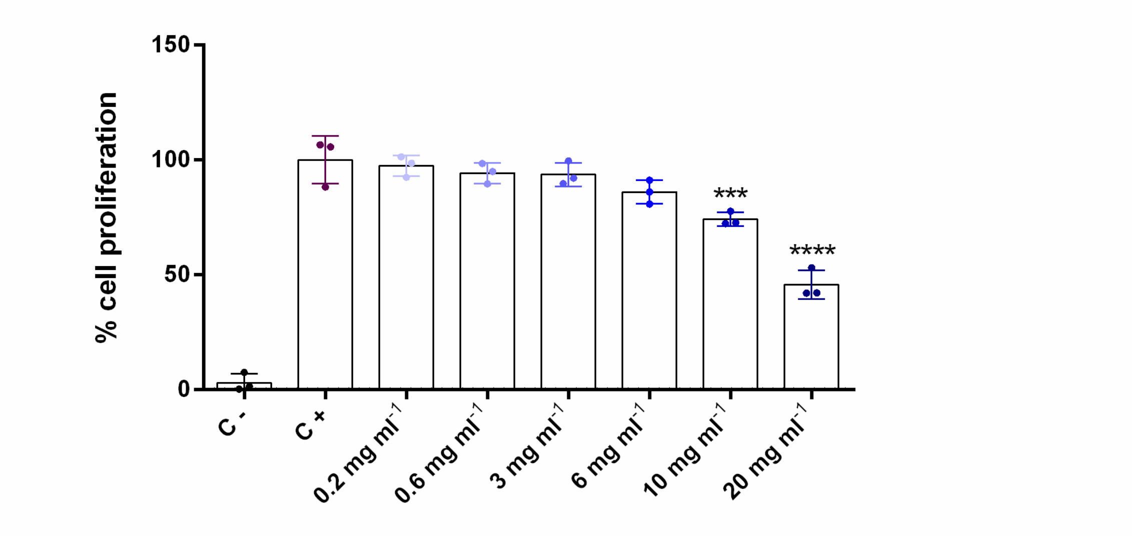

Results of MTS assay after 24 h of culture. 20,000 cells cm^{-2} of MSCs were seeded and their metabolic activity was measured after 24 h of incubation with different quantities of borax in the medium (0.2, 0.6, 3, 6, 10 and 20 mg mL^{-1}). The formation of formazan product was followed by measuring absorbance at 490 nm. All measurements were performed in triplicate from 3 different biological replicas. Statistics are shown as mean ± standard deviation. Data were analyzed by an ordinary one-way ANOVA test and corrected for multiple comparisons using Tukey´s analysis (P = 0.05). ***$p < 0.001$, ****$p < 0.0001$.

Supplementary Figure S2. Control of NaBC1 silencing and borax effects on focal adhesions (FA).

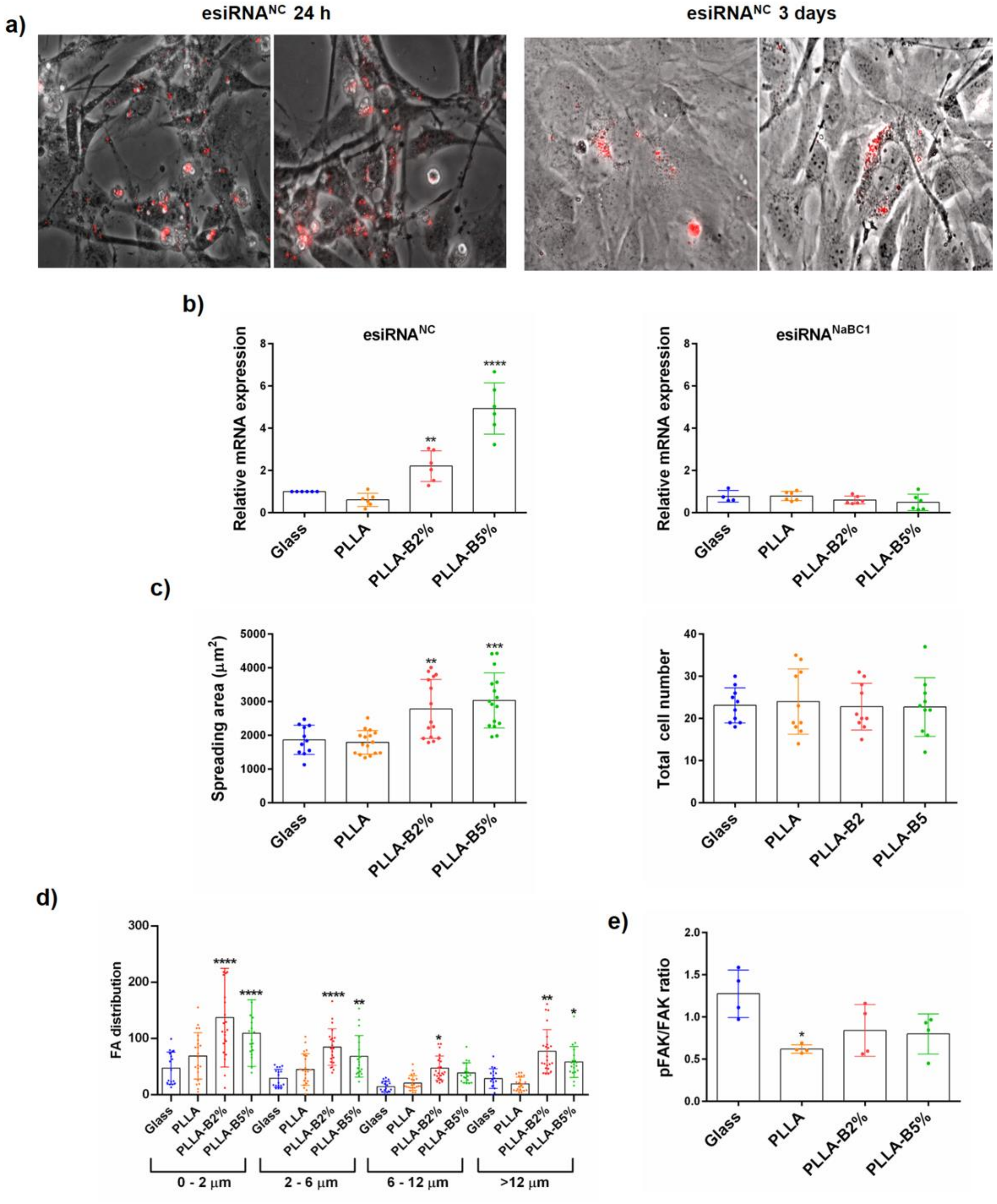

a) Microscopic images obtained after 24 h and 3 days of culture of transfected MSC cells with a universal negative control (esiRNA^{NC}) labelled with Cyanine 3 as a control for assessment of silencing efficiency.

b) qPCR amplification of NaBC1 mRNAs from transfected MSCs with esiRNA^{NC} and esiRNA^{NaBC1}. esiRNA^{NaBC1} transfected cells express reduced NaBC1 mRNAs confirming an efficient silencing of borate transporter. $n \geq 4$ different biological replicas.

c) Image analysis quantification of different parameters related to cell adhesion. Spreading area and total cell number. Borax induced higher cell spreading. 20 images/condition from three different biological replicas were analyzed. The data represented in graphs correspond to $n \geq 10$.

d) Focal adhesion distribution. Borax induced higher levels of nascent (0-6 µm) and mature FA (6-12 µm) formation. FA were counted from 20 images/condition from three different biological replicas.

e) In-Cell Western quantification of pFAK/FAK ratio. Borax induced FA formation is not dependent on FAK phosphorylation. n = 4 different biological replicas.

Statistics are shown as mean ± standard deviation. Data were analyzed by an ordinary one-way ANOVA test and corrected for multiple comparisons using Dunnett analysis (P = 0.05). $*p < 0.05$, $**p < 0.01$, $***p < 0.001$, $****p < 0.0001$.

Supplementary Figure S3. NaBC1 expression.

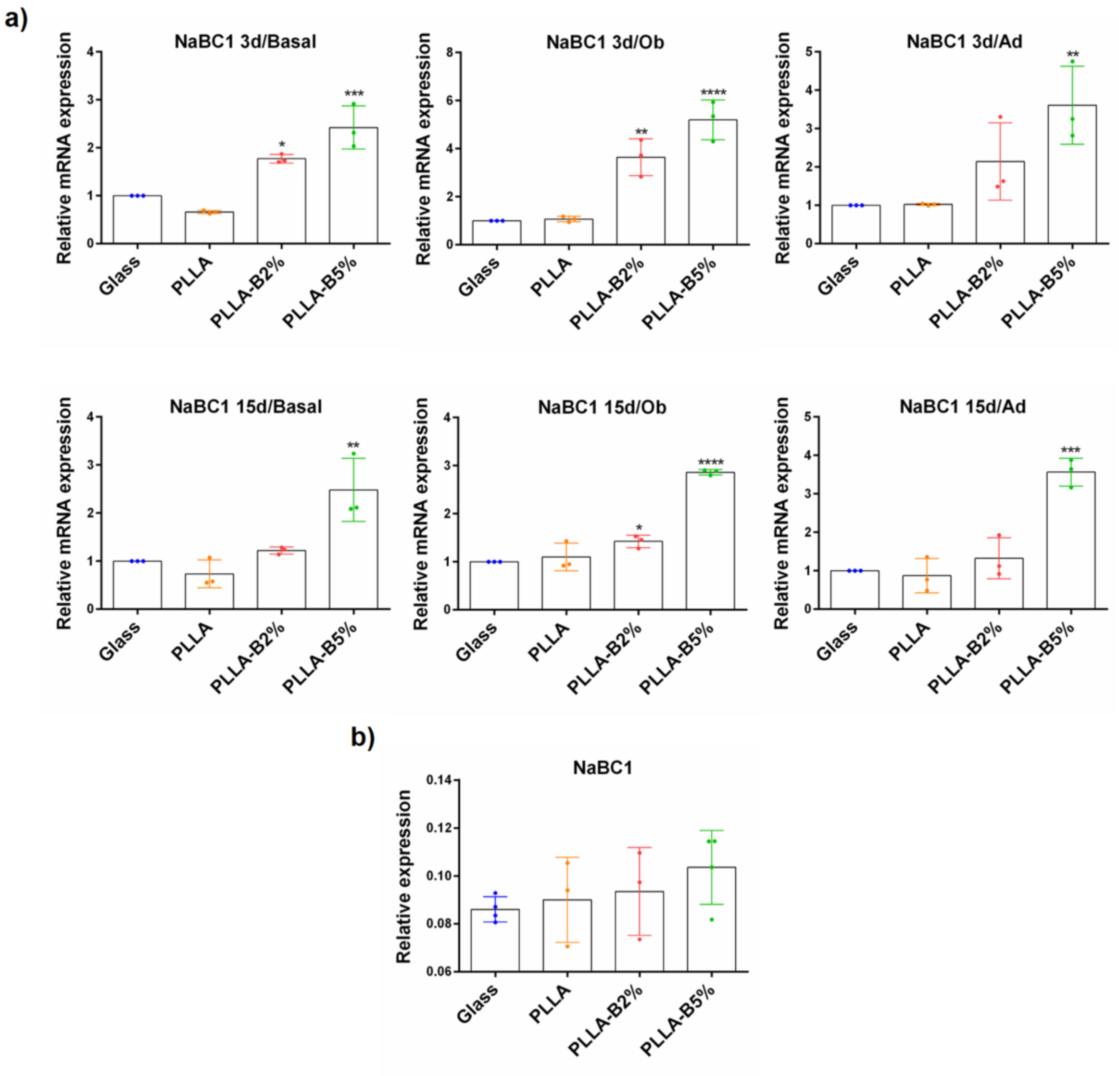

a) qPCR analysis of relative mRNA expression of borate transporter NaBC1. RNA was extracted from MSCs after 3 or 15 days of culture under both basal and differentiation conditions (osteogenic and adipogenic differentiation media). n = 3 different biological replicas.

b) In-Cell Western quantification of NaBC1. n ≥ 3 different biological replicas.

Statistics are shown as mean ± standard deviation. Data were analyzed by an ordinary one-way ANOVA test and corrected for multiple comparisons using Tukey analysis (P = 0.05). *p < 0.05, **p < 0.01, ***p < 0.001, ****p <0.0001.

Supplementary Figure S4. In-Cell Western quantification of FN-binding integrins and BMPR1A.

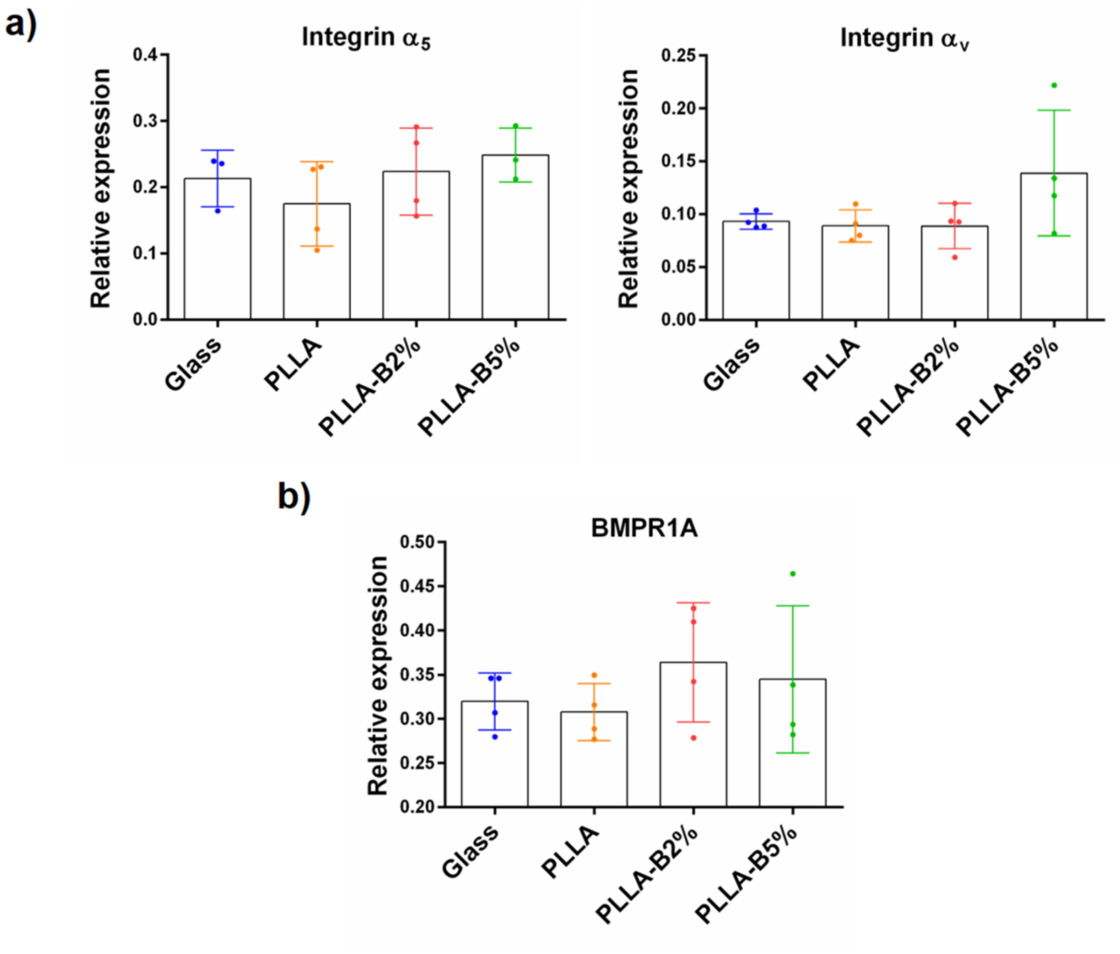

a) In-Cell Western quantification of $\alpha_{5,}$ α_v integrins. n ≥ 3 different biological replicas.

b) In-Cell Western quantification of BMPR1A. n = 4 different biological replicas.

Supplementary Figure S5: Borax effects on myosin light chain phosphorylation and actin stress fiber formation.

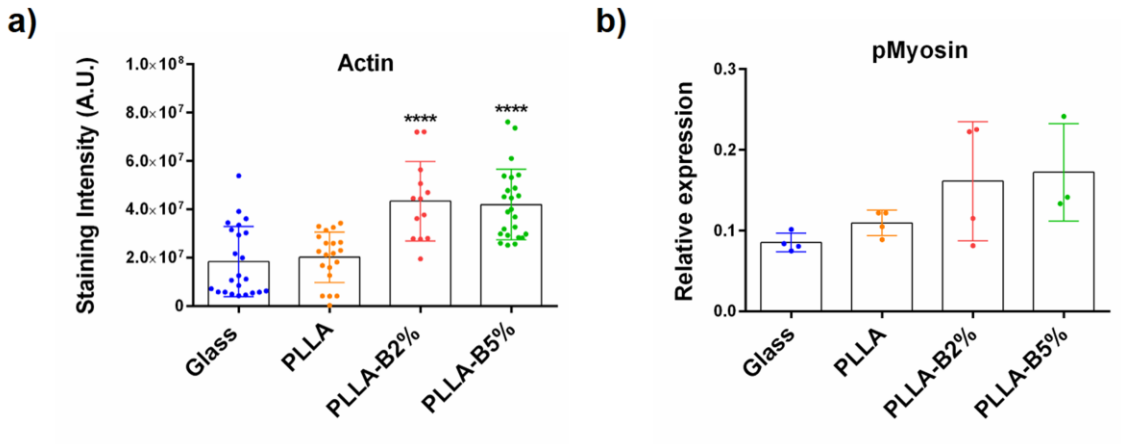

a) Image analysis quantification from images of Figure 4, of actin fibers as a parameter related to cell contractility. Active NaBC1 induces cell contractility. 30 images/condition from 3 different biological replicas were analyzed. The data represented in graphs correspond to $n \geq 13$.

b) In-Cell Western quantification of pMyosin. Active NaBC1 induce myosin light chain phosphorylation. $n = 4$ different biological replicas.

Statistics are shown as mean ± standard deviation. Data were analyzed by an ordinary one-way ANOVA test and corrected for multiple comparisons using Dunnett analysis ($P = 0.05$). ****$p < 0.0001$.

Supplementary Figure S6: Analysis of contractility using pharmacological inhibitors.

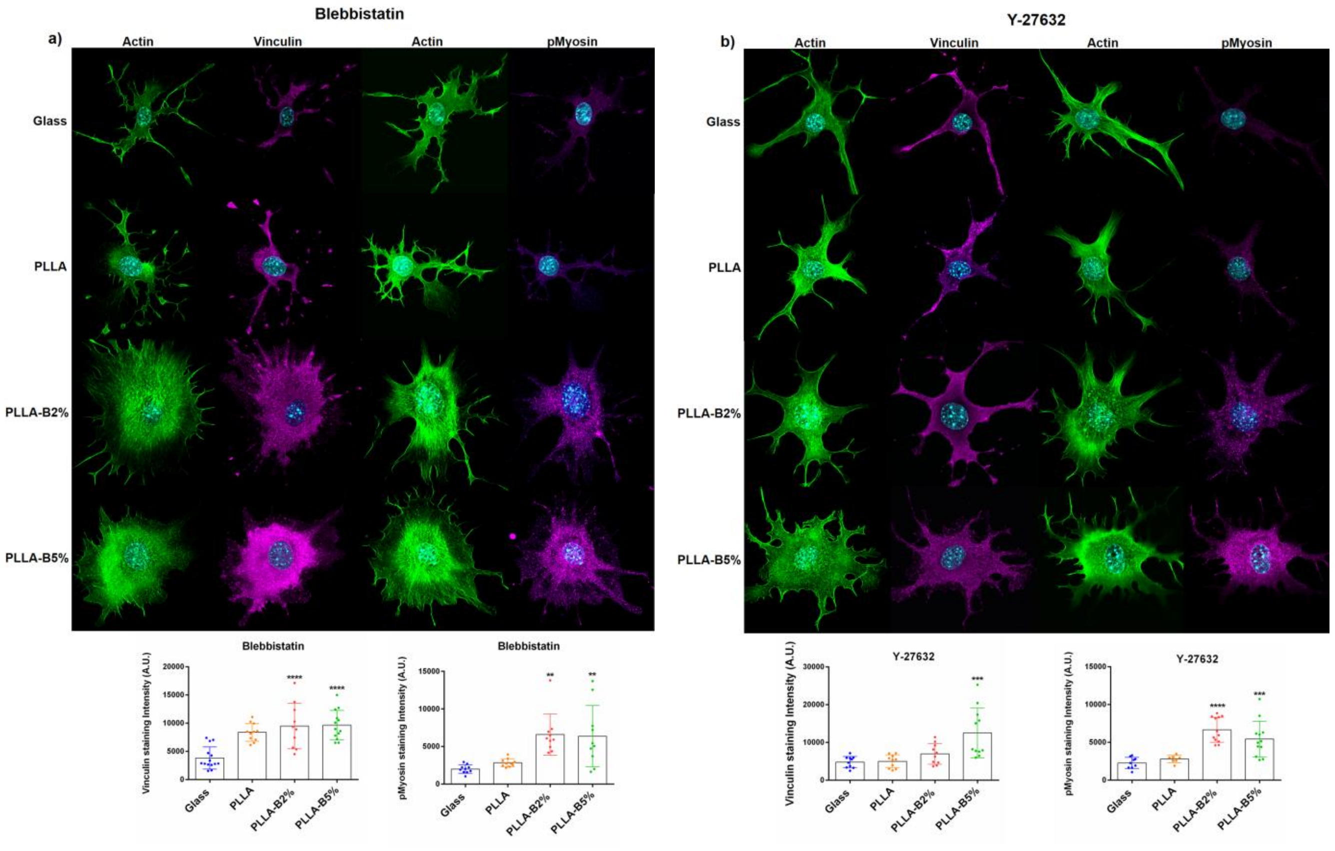

a) Immunofluorescence images of actin cytoskeleton (green), nuclei (cyan), vinculin and pMLC (pMyosin, magenta) after inhibition of contractility with Blebbistatin (Myosin II inhibitor). The PLLA-B2% and PLLA-B5% substrates presented higher levels of vinculin and pMyosin staining even after Blebbistatin addition compared to PLLA and Glass control substrates. ≥ 12 images/condition from three different biological replicas were analyzed. The data represented in graphs correspond to n ≥ 10.

b) Immunofluorescence images of actin cytoskeleton (green), nuclei (cyan), vinculin and pMLC (pMyosin, magenta) after inhibition of contractility with Y-27632 (Rho-kinase inhibitor). The PLLA-B2% and PLLA-B5% substrates presented higher levels of vinculin and pMyosin staining even after Y-27632 addition compared to PLLA and Glass control substrates. ≥ 12 images/condition from three different biological replicas were analyzed. The data represented in graphs correspond to n ≥ 10.

Statistics are shown as mean ± standard deviation. Data were analyzed by an ordinary one-way ANOVA test and corrected for multiple comparisons using Dunnett analysis (P = 0.05). **p < 0.1, ***p <0.001, ****p < 0.0001.

Supplementary Figure S7. Borax effects on MSC differentiation.

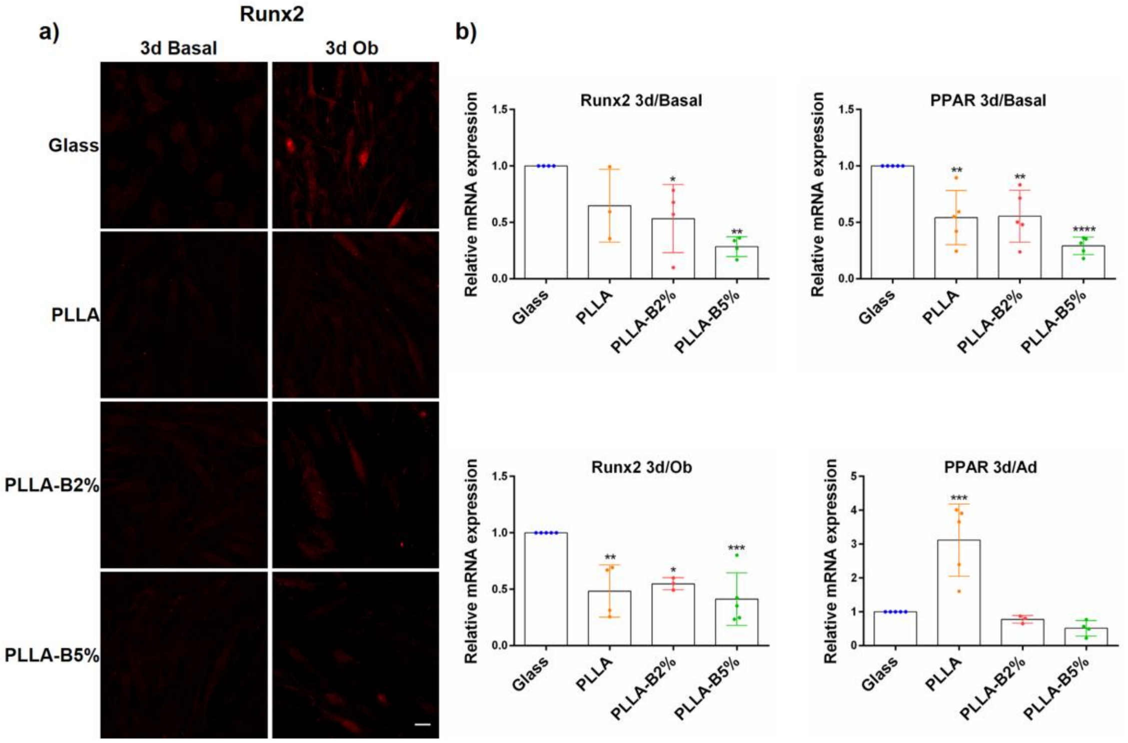

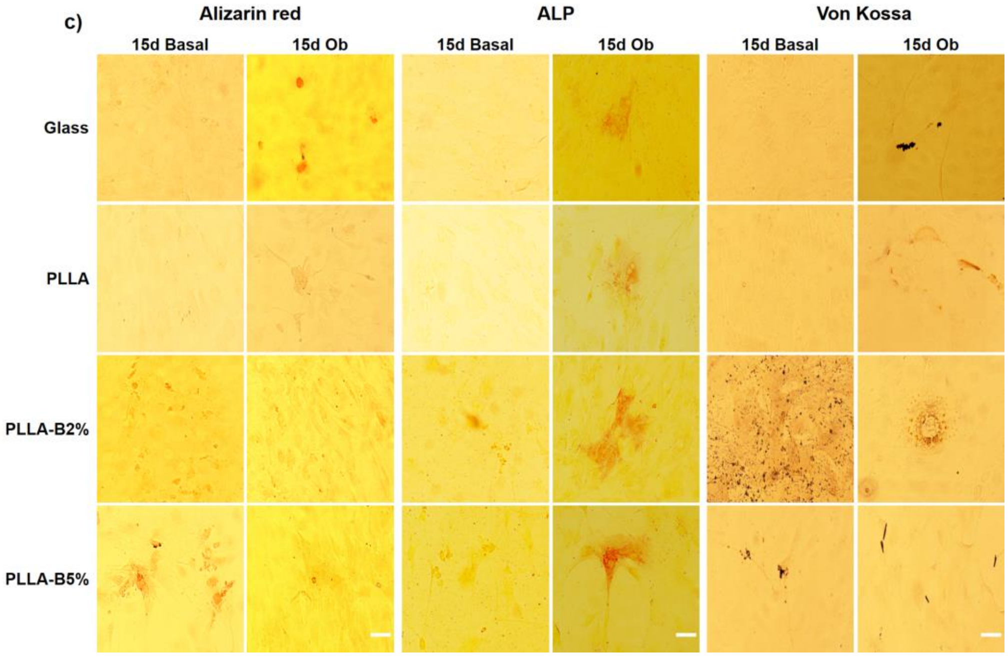

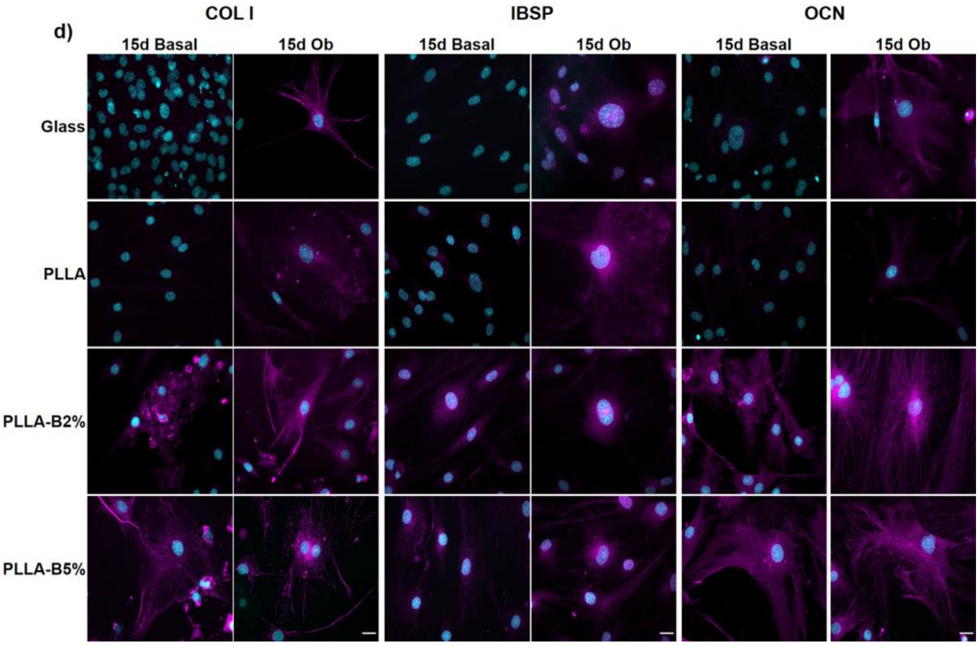

a) Immunofluorescence images of MSCs cultured for 3 days onto functionalized substrates (FN-coated) and borax (PLLA-B2%, PLLA-B5%) in culture medium, under basal and differentiation conditions (osteogenic), for detection of early osteogenic marker (Runx2, red). Scale bar 25 µm.

b) qPCR analysis of relative mRNA expression of early expressed transcription factors involved in osteogenic (Runx2) and adipogenic (PPARγ2) lineage determination. RNA was extracted after 3 days of culture under both basal and differentiation conditions (osteogenic and adipogenic differentiation media). $n \geq 3$ different biological replicas. Statistics are shown as mean ± standard deviation. Data were analyzed by an ordinary one-way ANOVA test and corrected for multiple comparisons using Dunnett analysis ($P = 0.05$). *$p < 0.05$, **$p < 0.01$, ***$p < 0.001$, ****$p < 0.0001$.

c) Histological staining of MSCs cultured for 15 days onto functionalized substrates (FN-coated) and borax (PLLA-B2%, PLLA-B5%) in culture medium, under basal and differentiation conditions (osteogenic), for detection of late osteogenic markers (Alizarin Red-calcium deposits in red, Alkaline Phosphatase activity (ALP) in red and Von Kossa-calcium deposits in black). Scale bar 25 µm.

d) Immunofluorescence images of MSCs cultured for 15 days onto functionalized substrates (FN-coated) and borax (PLLA-B2%, PLLA-B5%) in culture medium, under basal and differentiation conditions (osteogenic), for detection of late osteogenic markers: Collagen I (Col I), Integrin binding sialoprotein (IBSP) and Osetocalcin (OCN). Scale bar 25 µm. Borax alone induces osteogenic markers expression.
