# Supplementary material for: Borax induces osteogenesis by stimulating NaBC1 transporter via activation of BMP pathway
Source: Commun Biol. 2020 Nov 27;3:717. doi: 10.1038/s42003-020-01449-4 (PMC7695834; doi:10.1038/s42003-020-01449-4)
Supplement: Supplementary file 3 — Reporting Summary [file 42003_2020_1449_MOESM3_ESM.pdf]

nature research

Corresponding author(s): Patricia Rico, Manuel Salmeron-Sanchez

Last updated by author(s): 2020/10/18

Reporting Summary

Nature Research wishes to improve the reproducibility of the work that we publish. This form provides structure for consistency and transparency in reporting. For further information on Nature Research policies, see our Editorial Policies and the Editorial Policy Checklist.

Statistics

For all statistical analyses, confirm that the following items are present in the figure legend, table legend, main text, or Methods section.

n/a	Confirmed	
☐	☒	The exact sample size (n) for each experimental group/condition, given as a discrete number and unit of measurement
☐	☒	A statement on whether measurements were taken from distinct samples or whether the same sample was measured repeatedly
☐	☒	The statistical test(s) used AND whether they are one- or two-sided<br>*Only common tests should be described solely by name; describe more complex techniques in the Methods section.*
☒	☐	A description of all covariates tested
☐	☒	A description of any assumptions or corrections, such as tests of normality and adjustment for multiple comparisons
☐	☒	A full description of the statistical parameters including central tendency (e.g. means) or other basic estimates (e.g. regression coefficient) AND variation (e.g. standard deviation) or associated estimates of uncertainty (e.g. confidence intervals)
☐	☒	For null hypothesis testing, the test statistic (e.g. F, t, r) with confidence intervals, effect sizes, degrees of freedom and P value noted<br>*Give P values as exact values whenever suitable.*
☒	☐	For Bayesian analysis, information on the choice of priors and Markov chain Monte Carlo settings
☒	☐	For hierarchical and complex designs, identification of the appropriate level for tests and full reporting of outcomes
☒	☐	Estimates of effect sizes (e.g. Cohen's d, Pearson's r), indicating how they were calculated

Our web collection on statistics for biologists contains articles on many of the points above.

Software and code

Policy information about availability of computer code

Data collection	No software was used
Data analysis	Fiji-ImageJ (latest update), Graphpad Prism 8.0, CellProfiler 3

For manuscripts utilizing custom algorithms or software that are central to the research but not yet described in published literature, software must be made available to editors and reviewers. We strongly encourage code deposition in a community repository (e.g. GitHub). See the Nature Research guidelines for submitting code & software for further information.

Data

Policy information about availability of data

All manuscripts must include a data availability statement. This statement should provide the following information, where applicable:

- Accession codes, unique identifiers, or web links for publicly available datasets
- A list of figures that have associated raw data
- A description of any restrictions on data availability

Data has been uploaded to University of Glasgow's public data repository http://researchdata.gla.ac.uk/. DOI: 10.5525/gla.researchdata.1076

Field-specific reporting

Life sciences study design

All studies must disclose on these points even when the disclosure is negative.

Sample size	n >= 3 for all samples, detailed in each figure caption of the manuscript
Data exclusions	No data exclusions
Replication	All experiments were done at least in triplicate, in qPCR we used 3, 6 or 9 biological replicates
Randomization	Not relevant to our study
Blinding	Data processing was realized assigning a random number for each sample, and the researcher doing the analysis was not aware of the identity of the sample.

Reporting for specific materials, systems and methods

We require information from authors about some types of materials, experimental systems and methods used in many studies. Here, indicate whether each material, system or method listed is relevant to your study. If you are not sure if a list item applies to your research, read the appropriate section before selecting a response.

Materials & experimental systems

n/a	Involved in the study	
☐	☒	Antibodies
☐	☒	Eukaryotic cell lines
☒	☐	Palaeontology and archaeology
☒	☐	Animals and other organisms
☒	☐	Human research participants
☒	☐	Clinical data
☒	☐	Dual use research of concern

Methods

n/a	Involved in the study	
☒	☐	ChIP-seq
☒	☐	Flow cytometry
☒	☐	MRI-based neuroimaging

Antibodies

Antibodies used

Vinculin: mouse monoclonal, Sigma-Aldrich V9131, clone hVIN1
pMLC: mouse monoclonal, Cell Signaling 3675S
Integrin a5: rabbit monoclonal, Abcam ab150361, clone EPR7854
Integrin av: rabbit monoclonal, Abcam ab179475, clone EPR16800
Smad/pSmad1: rabbit monoclonal, Cell Signaling 12656
active YAP: rabbit monoclonal, Abcam ab205270, clone EPR19812
Runx2: mouse monoclonal, Abcam ab76956
FAK: anti rabbit, Millipore 06-543
pFAK: anti mouse, Millipore 05-1140
Osteopontin: mouse monoclonal, Santa Cruz Biotechnology sc-21742, clone AKm2A1
Osteocalcin: mouse monoclonal, Abcam ab13421, clone OCG4
Integrin binding sialoprotein: mouse monoclonal, Santa Cruz Biotechnology sc-73634, clone LMFb-24
Collagen I: mouse monoclonal, Abcam ab6308, clone COL-1
BMPR1A: rabbit polyclonal, Abcam ab38560,
NaBC1: goat polyclonal, Abcam ab99459
Runx2: anti mouse, Stratech ALS13287
pRunx2: anti rabbbit, 2B Scientific ARG54883
ERK: anti rabbit, Cell Signaling 4685
pERK: anti rabbit, Cell Signaling 4370
Akt/pAkt: rabbit polyclonal, Thermofisher PA1-22099
anti mouse Cy3 conjugated: Jacson Immunoresearch 115-165-003
anti mouse/rabbit Alexa fluor 555: Thermofisher A-21424/A-21429
anti mouse/rabbit Alexa fluor 488: Themofisher A-21042/A-11008

Validation

Integrin a5: Knockout validated by the manufacturer
active YAP: Knockout validated by the manufacturer

Eukaryotic cell lines

Policy information about cell lines

Cell line source(s)	Riken BRC Cell Bank, Japan
Authentication	Cells were authenticated in this publication Reznikoff, C A, Brankow, D W, Heidelberger C Establishment and characterization of a cloned line of C3H mouse embryo cells sensitive to postconfluence inhibition of division. Cancer Res 1973 33:3231-8 PubMed ID: 4357355
Mycoplasma contamination	Cell lines were not tested for mycoplasma contamination
Commonly misidentified lines (See ICLAC register)	None were used
